# The Progressive BSSG Rat Model of Parkinson's: Recapitulating Multiple Key Features of the Human Disease

**DOI:** 10.1371/journal.pone.0139694

**Published:** 2015-10-06

**Authors:** Jackalina M. Van Kampen, David C. Baranowski, Harold A. Robertson, Christopher A. Shaw, Denis G. Kay

**Affiliations:** 1 Neurodyn Inc., 550 University Ave, Charlottetown, Prince Edward Island, C1A 4P3, Canada; 2 Dept. Biomedical Science, University of Prince Edward Island, 550 University Ave, Charlottetown, Prince Edward Island, C1A 4P3, Canada; 3 Dept. Ophthalmology and Visual Sciences, University of British Columbia, Vancouver, British Columbia, Canada; UCL Institute of Neurology, UNITED KINGDOM

## Abstract

The development of effective neuroprotective therapies for Parkinson's disease (PD) has been severely hindered by the notable lack of an appropriate animal model for preclinical screening. Indeed, most models currently available are either acute in nature or fail to recapitulate all characteristic features of the disease. Here, we present a novel progressive model of PD, with behavioural and cellular features that closely approximate those observed in patients. Chronic exposure to dietary phytosterol glucosides has been found to be neurotoxic. When fed to rats, *β*-sitosterol *β*-d-glucoside (BSSG) triggers the progressive development of parkinsonism, with clinical signs and histopathology beginning to appear following cessation of exposure to the neurotoxic insult and continuing to develop over several months. Here, we characterize the progressive nature of this model, its non-motor features, the anatomical spread of synucleinopathy, and response to levodopa administration. In Sprague Dawley rats, chronic BSSG feeding for 4 months triggered the progressive development of a parkinsonian phenotype and pathological events that evolved slowly over time, with neuronal loss beginning only after toxin exposure was terminated. At approximately 3 months following initiation of BSSG exposure, animals displayed the early emergence of an olfactory deficit, in the absence of significant dopaminergic nigral cell loss or locomotor deficits. Locomotor deficits developed gradually over time, initially appearing as locomotor asymmetry and developing into akinesia/bradykinesia, which was reversed by levodopa treatment. Late-stage cognitive impairment was observed in the form of spatial working memory deficits, as assessed by the radial arm maze. In addition to the progressive loss of TH^+^ cells in the substantia nigra, the appearance of proteinase K-resistant intracellular α-synuclein aggregates was also observed to develop progressively, appearing first in the olfactory bulb, then the striatum, the substantia nigra and, finally, hippocampal and cortical regions. The slowly progressive nature of this model, together with its construct, face and predictive validity, make it ideal for the screening of potential neuroprotective therapies for the treatment of PD.

## Background

One of the greatest difficulties facing Parkinson's disease (PD) research is the inability to translate preclinical findings into meaningful clinical outcomes. While several putative neuroprotective compounds have been described in animal studies, no therapy has yet been identified that can definitively slow or stop the progression of PD. This is largely due to the lack of appropriate progressive rodent models [1]. The vast majority of PD animal models fail to mimic the progressive and protracted nature of the disease, which may be critical for the proper identification of neuroprotective therapies. Furthermore, there is no animal model capable of replicating all of the features of human idiopathic PD. While prodromal changes, such as olfaction, are not often observed in animal models [2, 3], this may be an important feature for screening neuroprotective strategies intended for early disease-modification. Changes in tyrosine hydroxylase (TH) and other dopamine markers do not always occur in transgenic mouse models, while aggregation of α-synuclein is only rarely seen in toxin models [4, 5]. Cognitive deficits are rarely observed and, when they are, tend to occur early and are not associated with histopathology typical of PD dementia.

Consumption of dietary neurotoxins derived from the seed of the cycad has been linked to the Guamanian neurological disease cluster ALS-parkinsonism dementia complex (ALS-PDC) in humans. When fed to rodents, cycad flour triggers the progressive development of neurological deficits, with behavioural and cellular features that closely approximate those observed in patients. Clinical signs and histopathology continue to develop for several months following cessation of exposure to the neurotoxic insult. [6–9]. Cycad-fed rats exhibit the progressive development of a neurological phenotype that is strikingly similar to Parkinson's disease. This includes the appearance of motor abnormalities, striatal dopamine loss, loss of dopaminergic neurons of the SNc, and Lewy body-like, ubiquitin-positive, α-synuclein aggregates [10, 11]. Clinical signs and histopathology continue to develop for several months following cessation of exposure to the neurotoxic insult.

## Objective

*In vitro* studies using isolated cycad compounds have demonstrated that several of these are neurotoxic. Specifically, a number of water insoluble phytosterol glucosides were found to have toxic properties. Of these, β-sitosterol β-D-glucoside (BSSG), which forms the largest fraction, is neurotoxic both *in vitro* and *in vivo* [8]. Here, we present an in-depth assessment of a novel, progressive rodent model of PD based on the oral consumption of BSSG. Assessment includes early prodromal features, locomotor deficits, cognitive dysfunction, the progressive loss of nigrostriatal neurons, and the regional spread of synuclein inclusions.

## Methods

### Animals

All studies used male Sprague Dawley rats (CD) (Charles River), approximately 3 months of age. Animals were housed in a specific pathogen free, temperature-controlled environment with a 12 h light/dark cycle and 24 hour *ad libitum* access to standard chow and water, except during BSSG intoxication, when *ad libitum* access was limited to 8 hours each day. Animals were individually housed in standard shoebox cages, with filter top lids and beta chip bedding. A PVC tube was placed in each cage for enrichment. Each experimental group contained 10 randomly assigned animals, based on a power analysis of earlier work with BSSG[12]. Experimental groups were treated and assessed using a balanced design in all procedural aspects, including animal husbandry. All animal experimentation was conducted in accordance with the CCAC guidelines for the care and use of laboratory animals and were approved by the University of Prince Edward Island institutional Animal Care Committee (ACC).

### BSSG intoxication

The toxin, *β*-sitosterol *β*-d-glucoside (BSSG), was prepared and administered in the form of a flour pellet. Five days a week for the first 4 months of the study, animals (*n* = 20) were randomly designated to receive either a plain flour pellet or one containing BSSG (3 mg/day). Pilot testing has revealed this to be more effectively than feeding BSSG a full 7 days a week. Animals were closely monitored to ensure complete pellet consumption. During BSSG feeding, animals were given ad libitum access to standard rat chow beginning two hours following BSSG pellet consumption and food was removed approximately 8 hours later (6 p.m.). This feeding schedule was intended to both motivate pellet consumption and maximize BSSG absorption.

### Behavioural analysis

At 4, 6, 8, and 10 months following initial BSSG exposure, animals underwent assessments for locomotor impairments. The same group of animals was tested at each time point to reduce inter-subject variability. These time points were chosen based on previous experience with both BSSG and cycad [10, 12]. Locomotor activity was measured for 1 hour using a home cage video tracking system (MED Associates Inc.), designed to record total ambulatory distance, time spent ambulatory, number of ambulatory events, average velocity, time spent at rest, rotational behaviour (measuring asymmetry) and rearing. All behavioural assessments were performed under blinded conditions in which, both the experimenter and the rater were unaware of experimental group assignments.

Locomotor coordination was assessed using a Foot Misplacement Apparatus (Columbus Instruments). Briefly, animals were trained for 3 days to traverse a horizontal ladder. The ladder is open on one end, with a bright light, and a dark enclosure is located on the other end. An electrified plate, located under the rungs of the ladder, serves to provide a mild electrical shock during training and to record foot slips during testing. Following 3 days of training, the total number of foot slips, along with travel time, were recorded.

In order to further validate the model and confirm dopaminergic involvement, we examined the ability of an anti-parkinsonian drug to reverse locomotor deficits in BSSG-fed animals. Thus, in a cohort of animals, we examined the ability of levodopa to reverse the locomotor deficits observed at 8 and 10 months following initial BSSG exposure. Twenty four hours following baseline testing, animals were administered levodopa (20 mg/kg, i.p.; Sigma) preceded (30 minutes) by the decarboxylase inhibitor, benserazide (15 mg/kg, s.c.; Sigma) and tested 30 minutes later for locomotor coordination and activity, as described above. Levodopa testing was limited to these two later time points in an effort to prevent the development of sensitization and to prevent any undo influence on model development.

Locomotor asymmetry was assessed in a separate cohort of animals at 4 and 10 months following initiation of BSSG exposure using a multichannel harness rotometer linked to a microcomputer (RotaCount, OmniTech). Animals were habituated to the test chambers for 15 min prior to testing. Animals were then injected with methamphetamine sulfate (2 mg/kg, i.p.; Sigma) and tested for 1 hour. The number and direction of each full 360° rotation was recorded.

We were interested to know whether olfactory deficits preceded locomotor deficits, as occurs in PD patients. Thus, we included an early time point for olfactory testing at 3 months following initial BSSG exposure. Olfactory testing was also performed at 4, 6, and 8 months in order to confirm the stability of this deficit. Olfactory sensitivity was assessed using a simple olfactory discrimination test, designed to measure sensitivity without the confounding effects of olfactory learning or memory. This was achieved by simply comparing the time spent exploring a vanilla solution versus time spent exploring a water-only solution. Vanilla was chosen based on pilot studies that showed it to trigger the strongest exploratory response (largest ratio) of 3 odours tested, as compared to water only. Briefly, 2" X 2" squares of filter paper were soaked with either vanilla (1:100) or distilled water (0.5 ml) and placed in a small perforated petri dish. One of each was placed on either end of an open field (see above), buried under the bedding. The location of each odour was randomized. Time spent in the vicinity of each scent was recorded for 5 minutes. Testing was repeated once each day for 3 days. Lack of detection was defined by equal investigation between the vanilla solution and the water control (ratio = 1). Sniffing was defined when the animal’s nose was located 1 cm or less from the odour.

Cognitive function was tested using two tasks, the radial arm maze (RAM) and the spontaneous alternation T-maze test. RAM testing was performed at the final 10 month time point. The requirement for food restriction and extensive daily training has the potential to alter the evolution of pathology, our primary interest, and thus precluded testing at multiple earlier time points. By contrast, the spontaneous alternation T-maze test requires no training or food-restriction, permitting testing at both an early and late time point. This permitted a glimpse into cognitive function in early and late stages without threatening to alter disease development. Thus, a T-maze spontaneous alternation task was used to assess cognition at 4 and 10 months following initial BSSG exposure. The classic version of the T‐maze, utilized here, assesses hippocampal‐dependent spatial learning ability without requiring food deprivation or training, both of which could have the potential to interfere with disease development. In this task, the animal was placed in the start arm, facing away from the center platform. The animal traverses the arm and then chooses to go down either the left or right arm. A choice was determined when all four paws were placed across the start of that arm. The animal was placed directly back into the start arm, and all steps repeated for a total of 10 trials. The overall alternation rate (percentage of responses in which the animal chose an alternate arm) and the percentage position bias (percentage of responding to the side of the maze preferred by that individual rodent) were recorded. At 10 months following initial toxin exposure, the last cohort of animals was tested for cognitive deficits using a radial arm maze (RAM). Thus, animals were placed on a food-restricted diet. Animals had free access to food 4 h at the same time each day, beginning immediately following the scheduled completion of each RAM trial. Following one week of food-restriction, animals underwent an adaptation phase using the RAM. Each animal was placed on the center platform and permitted to explore for 5 min. During this phase, the reward, Cheetos^®^ (~1cm^3^), was scattered throughout the RAM. Cheetos are a cheese-flavored, puffed cornmeal snack, highly desired by the animals. This reward has been found to provide optimal learning of the task in control animals. All animals were acclimated to the reward 4 days prior to training. Then, the reward was placed half-way down the arms chosen to be baited. Finally, the reward was placed at the end of the baited arms. Following 3 days of the adaptation phase, each animal was given one trial per day, five trials per week, for a total of 25 trials. The RAM consisted of 8 arms, of which 5 were baited with the reward. The baited and unbaited arms were chosen randomly for each rat and remained constant throughout the experiment. At the beginning of each trial, the animal was placed on the central platform and permitted to move throughout the maze until either all five rewards had been taken or 10 min had elapsed. Entries into unbaited arms and re-entries into previously visited arms were recorded as reference memory and working memory errors, respectively. For analysis, data were divided into 5 blocks of 5 trials each, and the scores averaged over each block.

### Immunohistochemistry

Animals were sacrificed by transcardial perfusion with 4% paraformaldehyde. Brains were removed and postfixed for 24 h in 4% paraformaldehyde followed by cryoprotection in 30% sucrose. Symmetrical 30 μm-thick sections were cut on a freezing microtome and stored in a Millonigs solution. The left hemisphere was marked with a notch to differentiate between hemispheres. Free-floating sections were incubated in 0.3% Triton X–100/Tris-buffered saline for 15 minutes, in blocking solution (3% goat serum/0.3% Triton X–100/Tris-buffered saline [TBS]) for 1 hour at room temperature, followed by the appropriate primary antibody at 4°C overnight. Primary antibodies included tyrosine hydroxylase (TH) (Chemicon MAB 318 and ab152, 1:10,000), dopamine transporter (DAT) (Chemicon MAB 369, 1:1000), Iba–1 (Abcam ab107159, 1:2000), grp78 (Abcam ab21685, 1:1200) and activated caspase–3 (AbCam ab2302, 1:500). For fluorescent visualization, sections were incubated with the respective secondary antibody conjugated to either Alexa 488, Alexa 594, or Alexa 350 (Molecular Probes). In between steps, sections were washed for 3 X 10 minutes in TBS. Sections were also assessed for proteinase K-resistant α-synuclein (BD Biosciences 610787, 1:1200). To this end, sections were pretreated with 10μg/ml proteinase K (Sigma) at 37°C for 20 minutes. Adjacent sections were labeled for α-synuclein without proteinase K pretreatment, to assess total synuclein and confirm specificity. To assess ubiquitin co-labeling, these sections were also incubated with the primary antibody for ubiquitin (1:2000, rabbit anti-ubiquitin, AbCam). Nissl was assessed in free-floating sections using NeuroTrace (Invitrogen, 1:50). For anlaysis of beta-sheet formations, some sections were incubated in 1% Thioflavin-S (Sigma) for 20 minutes followed by 70% ethanol for 5 minutes, and several washes of distilled water. Sections were mounted on unsubbed glass slides and coverslipped in Fluoromount. For the *in situ* terminal deoxynucleotidyl transferease dUTP nick end labeling (TUNEL) assay (R&D Systems), sections were first mounted on clean glass slides and processed according to the manufacturer instructions. Fluorescence signals were detected with a Zeiss AxioObserver Z1 Imaging microscope equipped with an apotome system at excitation/emission wavelengths of 535/565 nm, 470/505 nm, and 585/615 nm. Slides were coded to render the experimenter blind to experimental group assignment.

### Image Analysis

Nigrostriatal integrity was determined by TH^+^ cell counts in the SN_C_ and striatal DAT immunodensity. As TH^+^ cell counts are only a marker of dopaminergic phenotype and do not necessarily imply cell death/survival, lesion severity was determined by assessment of both TH^+^ and Nissl^+^ cell counts. Stereological analyses of TH-positive and Nissl-positive neurons in the SNc were carried out by a blind experimenter using the optical fractionator method, an unbiased method of cell counting that is independent of the volume of the brain area considered and the size of neurons being counted. The SNc was delineated at 10x magnification using the Paxinos and Watson atlas for anatomical landmarks. TH^+^ neurons were counted from every 12^th^ serial section (30 μm) using a Zeiss AxioObserver Z1 Imaging microscope equipped with an apotome system for z stacking, area estimation and 3D reconstruction. The microscope was interfaced to a Dell Dimension 8100 workstation with Axiovision software. A counting frame of x = 100 μm and y = 100 μm was incorporated along with steps involving movements of x = 140 μm and y = 140 μm. For denstiometric analyses of striatal DAT, a sampling frame of x = 50 μm, y = 50 μm, and z = 20 μm was used to sample the dorsomedial, dorsolateral and ventrolateral regions of each of 3 sections through the striatum using Axiovision software. This approach was also used for densitometric analyses of synaptophysin immunolabeling in the hippocampus and cortex. Proteinase K-resistant α-synuclein aggregates were counted in the olfactory bulb, striatum, SNc, cortex, entorhinal cortex, and hippocampus using a sampling frame of x = 50 μm, y = 50 μm, and z = 20 μm. Aggregates were counted in every 12^th^ section, across 4 sections through each region. For grp78, we examined the density of grp78 immunolabeling within TH^+^ neurons of the SNc. To do this, we located a TH^+^ neuron, imaged it in 3 dimensions, and obtained densitometric measurements from the center 1.5 μm layer of the cell using a 5μm X 5μm sampling frame. Ten samples were obtained for each of 4 anterior-posterior serial sections through the SNc. For all assessments of the SNc, cells were counterstained with TH for accurate localization. Images remained coded during image analysis to ensure unbiased assessment.

### Statistical Analysis

Data were analyzed using a multivariant analysis of variance. Where significant F-values were obtained, planned pair-wise comparisons were made using Newman-Keuls. Differences were considered statistically significant when *p* < 0.05.

## Results

### Weight gain

At no time was the overall health of the animal negatively affected by BSSG feeding, and the animals did not show signs of distress. No BSSG-fed animals were lost in the course of this study, though two flour control animals were eliminated from the study after developing a small tumour. BSSG treatment did not induce weight loss. In fact, over time, BSSG-treated animals tended to gain slightly more weight than controls, with final weights being slightly higher in BSSG-fed animals (S1 Fig). This is consistent with our previous experience with this model [12].

### Early olfactory deficits

**Fig 1:**
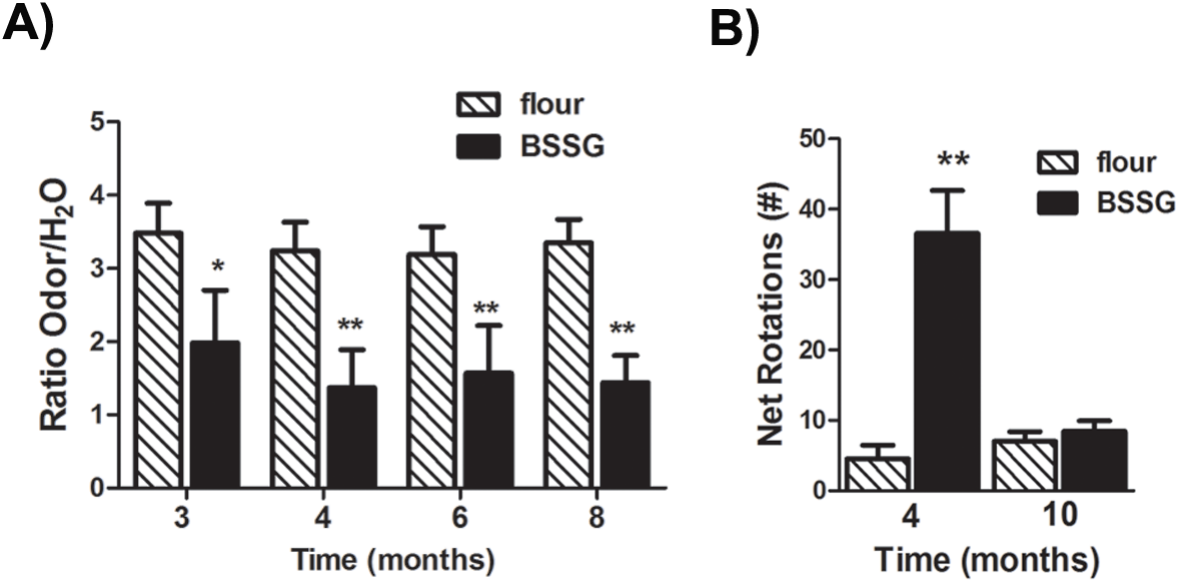
BSSG triggers early olfactory deficits and locomotor asymmetry. **(A)** Olfactory sensitivity was assessed by comparing the time spent exploring a vanilla solution versus time spent exploring a water-only solution. Lack of detection was defined by equal investigation between the odour solution and the water control (ratio = 1). Control animals spent more time in the vicinity of the odour, as compared to water alone. As early as 3 months following initiation of BSSG intoxication, animals spent a significantly smaller proportion of their time exploring the odour and, by 8 months, displayed little evidence of discrimination. Each bar represents the mean (± S.E.M., *n* = 18–20) ratio of exploration time. (**B)** At 4 months following initiation of BSSG exposure, animals displayed locomotor asymmetry, as assessed by methamphetamine (2 mg/kg, i.p.)-induced rotations. No significant increase in drug-induced rotations was observed 10 months following initial BSSG exposure. Each bar represents the mean (± S.E.M., *n* = 10) net rotations recorded in 1 hour. ** sig. diff. from flour control, *p* < 0.001, * *p* < .05.

Among a variety of non-motor symptoms observed in PD, hyposmia typically precedes the onset of motor features in PD in approximately 70–95% of patients [13, 14]. Here, animals were tested for olfactory sensitivity using a simple olfactory detection protocol that did not require extensive training. Using this protocol, we found that the ratio of time spent exploring an odour versus plain drinking water was significantly reduced in BSSG-fed animals across all time points, beginning as early as 3 months following initial exposure to the toxin (F_1,39_ = 649.72, *p<*0.0001) (Fig 1A). Control animals did not display any variability across the time points studied. These data suggest an early and persistent deficit in olfactory sensitivity.

### Locomotor dysfunction

**Fig 2:**
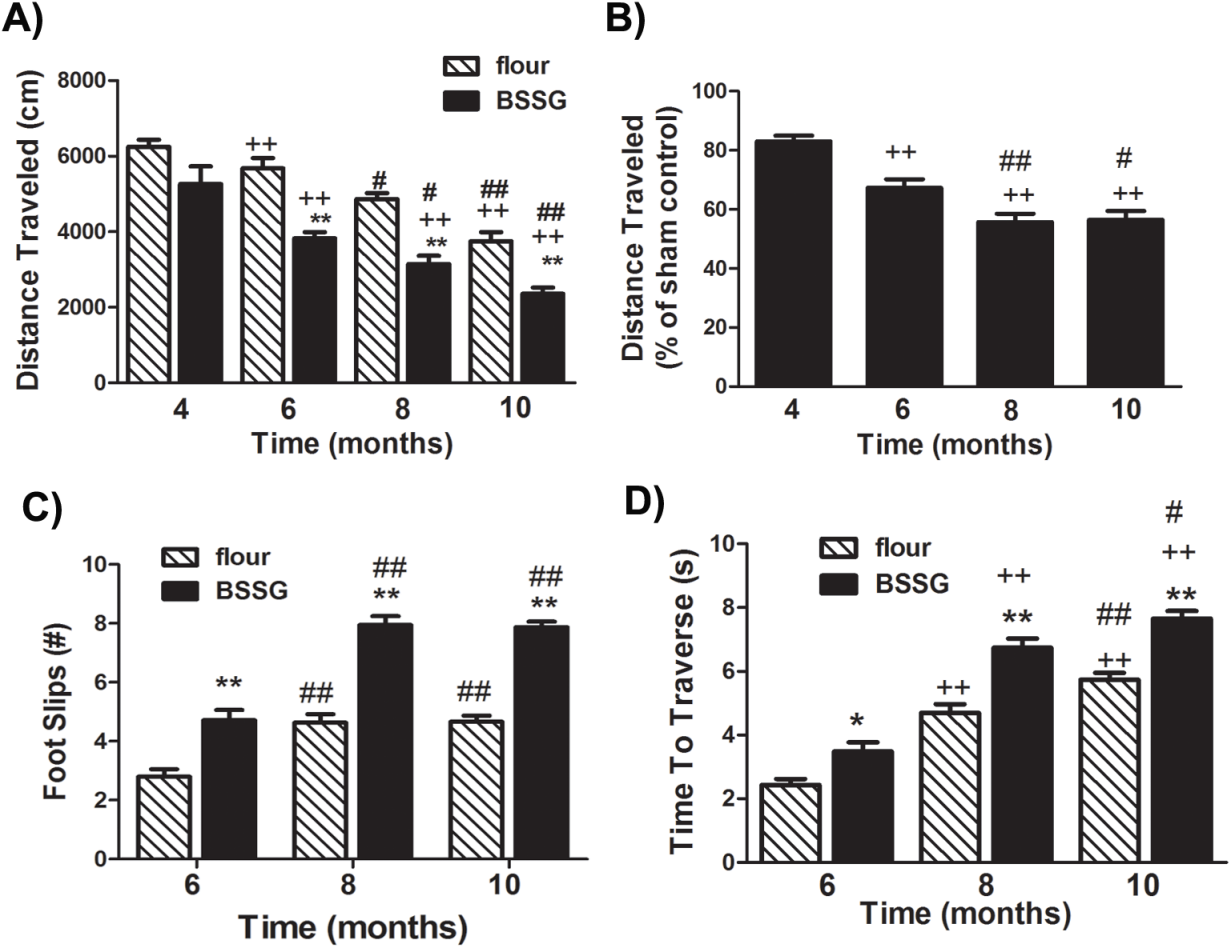
BSSG significantly reduced locomotor activity and coordination. **(A)** As early as 6 months following initiation of BSSG exposure, animals displayed a time-dependent reduction in locomotor activity, as assessed by the distance travelled over a 1 hour period. (**B)** Locomotor activity was significantly reduced to approximately 82%, 67%, 56%, and 56% of sham controls, at 4, 6, 8, and 10 months following initiation of BSSG exposure, respectively. Each bar represents the (**A)** mean (± S.E.M., *n* = 18–20) distance traveled in 1 hour or (**B)** mean (± S.E.M., *n* = 18–20) percent of sham control. (**C)** At 6, 8, and 10 months following initiation of BSSG exposure, animals displayed progressive deficits in locomotor coordination, as assessed by the number of foot slips when tested in the foot misplacement apparatus. The total number of foot slips was significantly greater than sham controls at all three time points. Each bar represents the mean (± S.E.M., *n* = 18–20) number of foot slips recorded. (**D)** Animals were also progressively slower to traverse the horizontal ladder in the foot misplacement apparatus. Each bar represents the mean (± S.E.M., *n* = 18–20) time (sec.) to traverse the ladder. ** sig. diff. from flour control, *p* < 0.001; ++ sig. diff. from 4 months, *p* < 0.001; ## sig. diff. from 6 months, *p*< 0.001; # *p*< 0.05.

Locomotor deficits first appeared in the form of locomotor asymmetry. Animals were challenged with the dopamine releasing agent, methamphetamine (2 mg/kg, i.p.), which triggered a rotational response. Animals treated with BSSG displayed significantly more net rotations than controls at 4 months following initial toxin exposure (F_1,35_ = 188.59, *p*<0.0001, BSSG main effect; F_1,35_ = 124.89, *p*<0.0001, time main effect; F_1,35_ = 188.29, *p*<0.0001, interaction effect) (Fig 1B). Following post-mortem analysis, it was confirmed that, observed rotations occurred ipsilateral to the hemisphere of greatest striatal atrophy. However, no significant increase in net rotations was observed at 10 months, suggesting that locomotor asymmetry is an early feature of this model, with motor deficits progressing to become bilateral over time. In an open field, locomotor activity, measured as the distance traveled in one hour, was significantly reduced, beginning as early as 6 months following initial toxin exposure, as compared to controls (F_1,38_ = 98.75, *p*<0.0001, BSSG main effect; F_3,117_ = 68.57, *p*<0.0001, time main effect; F_3,117_ = 3.53, *p* = 0.0173, interaction effect) (Fig 2A). This deficit worsened over time, with significantly less locomotor activity observed at 10 months, as compared to 6 months. There appeared to be a slight age-related decrease in locomotor activity, overall, and this decline was significantly potentiated in BSSG-treated animals (Fig 2B). Locomotor coordination was also impaired following BSSG exposure. BSSG-treated animals displayed significantly more foot slips while traversing a horizontal ladder (F_1,39_ = 164.19, *p*<0.0001, BSSG main effect; F_2,80_ = 29.44, *p*<0.0001, time main effect; F_2,80_ = 5.37, *p* = 0.0066, interaction effect) (Fig 2C). This deficit first appeared at 6 months following initial toxin exposure and significantly worsened by 10 months. The time to traverse the ladder also increased following BSSG exposure (F_1,39_ = 83.39, *p*<0.0001, BSSG main effect; F_2,80_ = 108.96, *p*<0.0001, time main effect; F_2,80_ = 2.96, *p*<0.0001, interaction effect) (Fig 2D).

**Fig 3:**
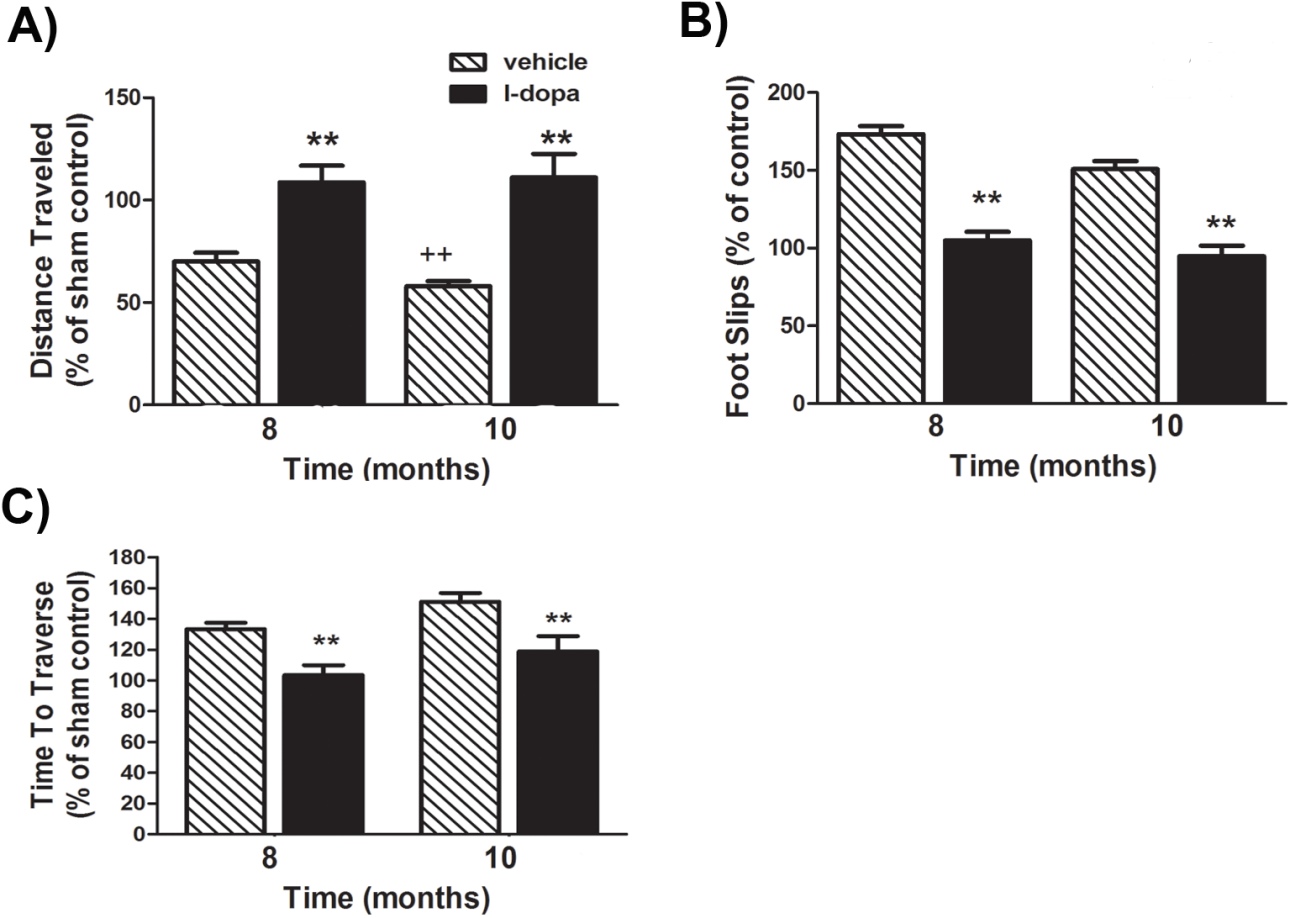
BSSG-induced locomotor deficits are corrected by the anti-parkinsonian drug, levodopa. **(A)** At 8 and 10 months following initiation of BSSG exposure, animals displayed a time-dependent reduction in locomotor activity, as assessed by the distance travelled over a 1 hour period. Locomotor activity was reduced to approximately 70% and 58%, respectively. Administration of the anti-parkinsonian drug, l-dopa (20 mg/kg, i.p.), preceded by the decarboxylase inhibitor, benserazide (15 mg/kg, s.c.), significantly reversed locomotor deficits, bringing locomotor activity back to control levels. Each bar represents the mean (± S.E.M., *n* = 18–20) distance traveled, expressed as a percentage of sham controls. (**B)** Administration of l-dopa also reversed deficits in locomotor coordination, as assessed by the number of foot slips when tested in the foot misplacement apparatus. Each bar represents the mean (± S.E.M., *n* = 18–20) foot slips, expressed as a percentage of sham controls. (**C)** Animals also required significantly more time to traverse the horizontal ladder at 8 and 10 months, an impairment reversed by l-dopa administration. Each bar represents the mean (± S.E.M., *n* = 18–20) time to traverse the ladder, expressed as a percentage of sham controls. ** sig. diff. from baseline control, *p* < 0.001; ++ sig. diff. from 8 months, *p* < 0.001; + *p* < 0.05.

Dopamine replacement therapy, in the form of levodopa/carbidopa, remains the most effective treatment for the motor symptoms of PD. Here, we examined the ability of levodopa to reverse the locomotor deficits observed at 8 and 10 months following initial BSSG exposure. Administration of the antiparkinsonian drug, levodopa (20 mg/kg, i.p.), preceded (30 minutes) by the decarboxylase inhibitor, benserazide (15 mg/kg, s.c.), significantly reversed BSSG-induced deficits in locomotor activity, measured in the open field (F_1,39_ = 282.31, *p*<0.0001, levodopa main effect; F_1,40_ = 0.3697, *p* = 0.5488, time main effect; F_1,40_ = 14.34, *p* = 0.0005, interaction effect) (Fig 3A). Following levodopa, there were no significant differences between BSSG-treated animals and controls in distance traveled. Levodopa treatment also reversed BSSG-induced deficits in locomotor coordination (F_1,39_ = 321.43, *p*<0.0001, levodopa main effect; F_1,40_ = 20.37, *p*<0.0001, time main effect; F_1,40_ = 4.44, *p* = 0.0418, interaction effect) (Fig 3B and 3C). Following levodopa treatment, no significant differences were observed in the number of foot slips or time taken to traverse the horizontal ladder between BSSG-treated animals or their controls.

### Cognitive deficits

**Fig 4:**
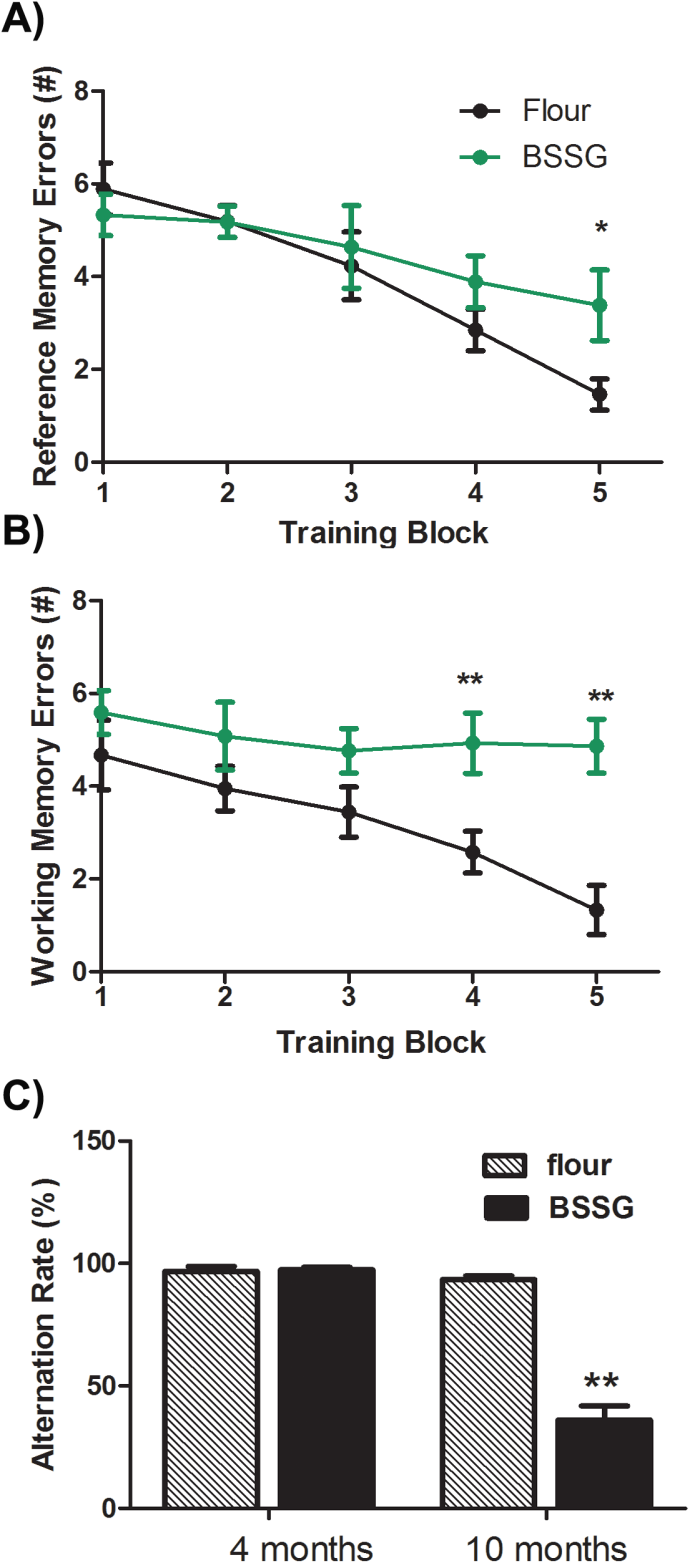
BSSG intoxication impairs spatial working memory. At 10 months following initiation of BSSG exposure, animals displayed significant impairment in both (**A)** reference and (**B)** working spatial memory, as assessed by their performance in a radial arm maze. Reference memory errors were defined as entries into arms that were never baited. Working memory errors were defined as entries into previously visited arms. Each point represents the mean (± S.E.M., *n* = 18–20) number of errors. (**C)** Spontaneous alternation in a T-maze was also impaired 10 months following initial BSSG exposure, as compared to controls. However, no such deficit was yet apparent at 4 months. ** sig. diff. from sham control, *p*< 0.001; * *p*<0.05.

While locomotor dysfunction is the most prominent feature of PD, non-motor features, such as dementia, are often more problematic. The majority of PD patients will develop dementia within 12 years [15], which is the primary cause of institutionalization of these patients. Here, BSSG intoxicated animals were tested for the development of cognitive deficits Animals exposed to BSSG did display some signs of cognitive impairment. At 4 and 10 months following initiation of BSSG exposure, animals were tested for spontaneous alternation in a T-maze (Fig 4C). Spontaneous alternation is considered a test of short-term working memory dependent on hippocampus, septum, basal forebrain, and prefrontal cortex [16]. Since this test does not require training or food-restriction, testing could be performed at both early and late time points without concern of influencing pathological development or behavioural response. Using this test, animals showed no cognitive impairment at 4 months but did show a significant impairment at 10 months following initial BSSG exposure (F_1,39_ = 433.92, *p*<0.0001, BSSG main effect; F_1,40_ = 559.45, *p*<0.0001, time main effect; F_1,40_ = 575.67, *p*<0.0001, interaction effect). At this time point, BSSG-intoxicated animals also displayed significant deficits in both reference and spatial working memory, as assessed by the eight arm radial arm maze (F_1,37_ = 38.89, *p*<0.0001, BSSG main effect; F_4,162_ = 295.26, *p*<0.0001, time main effect; F_4,152_ = 42.56, *p*<0.0001, interaction effect) (Fig 4A and 4B). Using this test, BSSG-fed animals were more likely to enter arms that were never baited. They also were more likely to re-enter arms they had already visited.

### Nigrostriatal integrity

**Fig 5:**
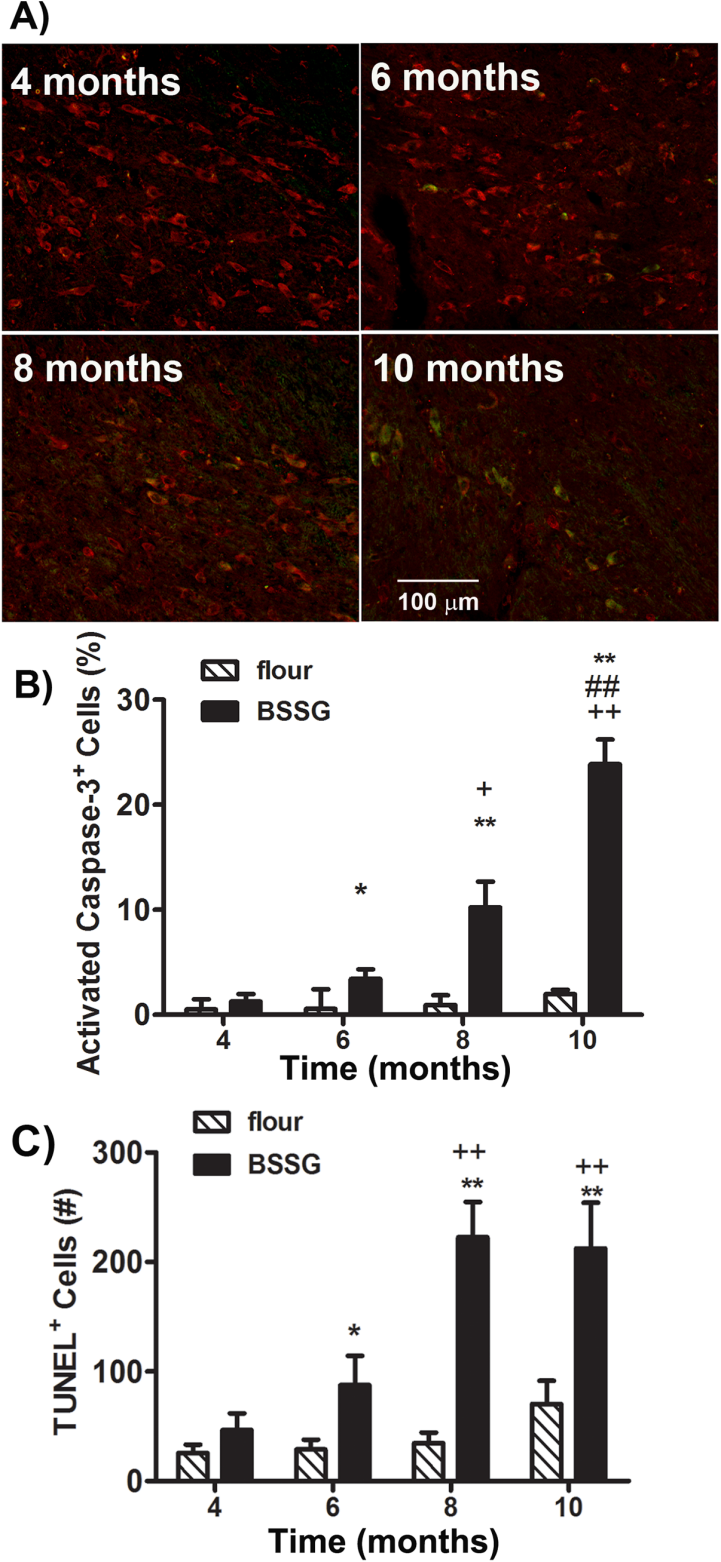
BSSG increases indices of apoptosis. **(A)** Representative photomicrographs of TH (red) and activated caspase–3 (green) immunostaining in the SNc 4, 6, 8, and 10 months following initial BSSG exposure. (**B)** Exposure to BSSG triggered the progressive increase in the percentage of TH+ cells expressing activated caspase–3, an indicator of apoptosis. Each bar represents the mean (± S.E.M., *n* = 9–10) percentage of TH+ cells labeled for activated caspase-3-positive cells in the SNc. (**C)** BSSG similarly triggered time-dependent elevations in TUNEL labeling within the SNc. Each bar represents the mean (± S.E.M., *n* = 9–10) number of TUNEL-positive cells counted in the SNc. ** sig. diff. from flour control, *p* < 0.001; * *p* < 0.05; ++ sig. diff. from 4 months, *p* < 0.001; *+ p* < 0.05; ## sig. diff. from 6 months, *p* < 0.001.

**Fig 6:**
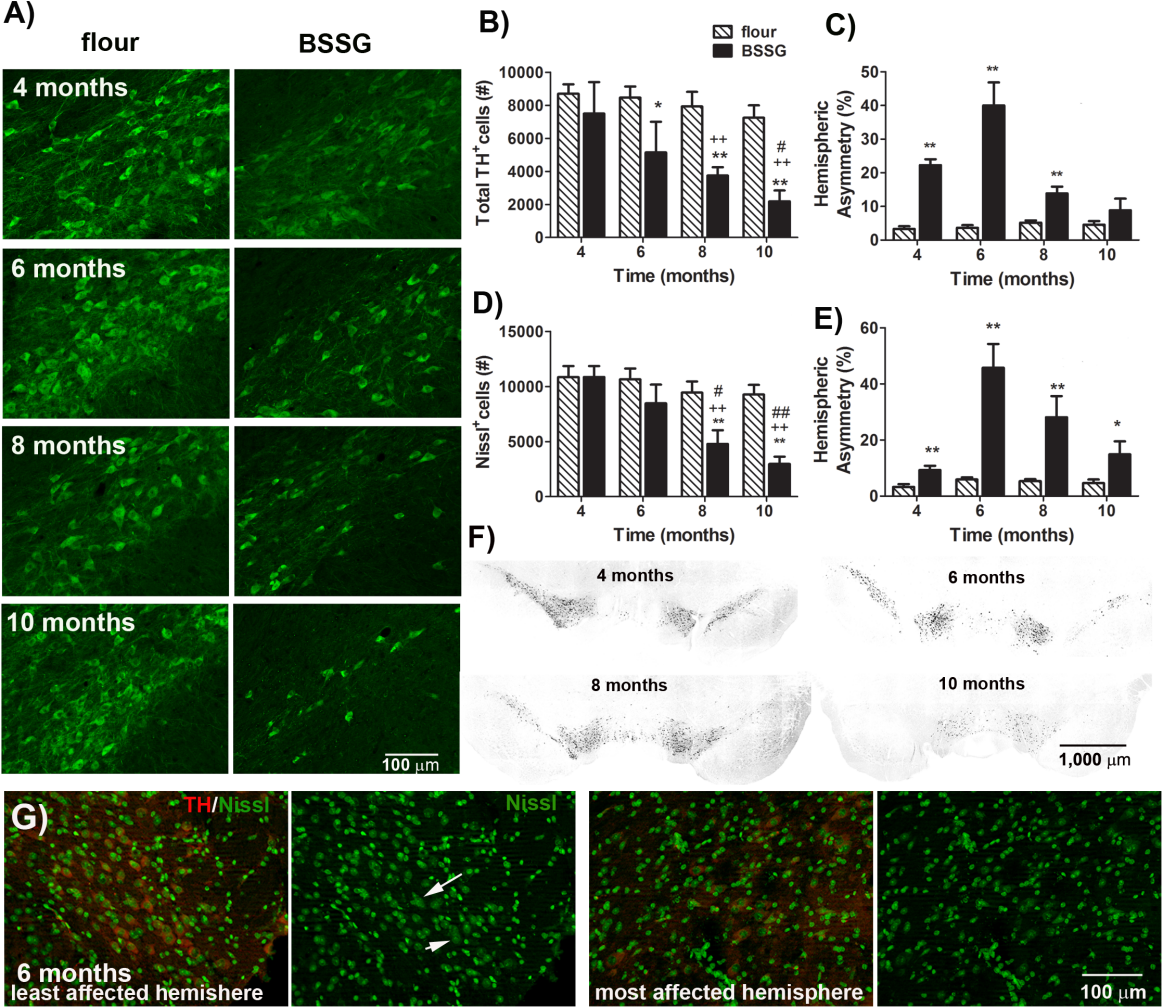
BSSG triggers the progressive loss of dopaminergic nigrostriatal neurons. **(A)** Representative fluorescent photomicrographs of TH immunostaining in the SNc 4, 6, 8, and 10 months following initial BSSG exposure. Unbiased stereologic counts of (**B)** TH^+^ and (**D)** Nissl^+^ cells in the SNc were significantly reduced in those animals treated with BSSG. Each bar represents the mean (± S.E.M., *n* = 9–10) number of TH or Nissl immunopositive cells counted in the SNc. Asymmetrical loss of both (**C)** TH^+^ and (**E)** Nissl^+^ cells in the SNc was observed. Each bar represents the mean (± S.E.M., *n* = 9–10) hemispheric difference, expressed as a percentage of the most populated hemisphere. (**F**) Representative photomicrographs depicting asymmetry of TH immunolabeling in the SNc 4, 6, 8, and 10 months following initial BSSG exposure. (**G**) Representative fluorescent photomicrographs depicting TH (red) and Nissl (green) labeling in both hemispheres of the SNc 6 months following initial exposure to BSSG. Arrows indicate Nissl-positive neurons. ** sig. diff. from flour control, *p* < 0.001; * *p* < 0.05; ++ sig. diff. from 4 months, *p* < 0.001; + *p* < 0.05; ## sig. diff. from 6 months, *p* < 0.001; # *p* < 0.05.

Animals were sacrificed by perfusion at 4, 6, 8, and 10 months following initial exposure to BSSG and their brains harvested for histopathological assessment of markers for cell death, nigrostriatal integrity, inflammation, and proteinase K-resistant synuclein deposits. Immunolabeling for activated caspase–3 and TUNEL, both apoptotic markers, progressively increased with time in the SNc of those animals exposed to BSSG, beginning at 6 months following initial exposure to the toxin (caspase: F_1,71_ = 233.44, *p*<0.0001, BSSG main effect; F_3,71_ = 65.53, *p*<0.0001, time main effect; F_3,71_ = 33.69, *p*<0.0001, interaction effect) (TUNEL: F_1,71_ = 252.34, *p*<0.0001, BSSG main effect; F_3,71_ = 56.21, *p*<0.0001, time main effect; F_3,71_ = 37.31, *p*<0.0001, interaction effect) (Fig 5). The persistence of this response, as opposed to the early rise and decline observed in many acute models, may be reflective of ongoing degeneration. In order to determine nigro-striatal integrity, sections were assessed for TH and dopamine transporter (DAT) immunolabeling in the SNc and striatum. As TH^+^ cell counts are only a marker of dopaminergic phenotype and do not necessarily imply cell death/survival, lesion severity was determined by assessment of both TH^+^ and Nissl cell counts. Stereological analyses of TH^+^ and Nissl^+^ neurons in the SNc revealed a significant loss of both following BSSG intoxication (TH: F_1,71_ = 185.56, *p*<0.0001, BSSG main effect; F_3,71_ = 16.15, *p*<0.0001, time main effect; F_3,71_ = 8.81, *p*<0.0001, interaction effect) (Nissl: F_1,71_ = 186.54, *p*<0.0001, BSSG main effect; F_3,71_ = 59.78, *p*<0.0001, time main effect; F_3,71_ = 29.96, *p*<0.0001, interaction effect) (Fig 6). However, significant nigral cell loss was not observed until after BSSG exposure was terminated. Thus, at 4 months following initial exposure to BSSG, when toxin feeding was terminated, there was no significant loss of either TH^+^ or Nissl^+^ cells in the SNc. However, a time-dependent, progressive loss of these cells was observed in the ensuing months, with nearly 70% fewer TH^+^ cells observed in the SNc by 10 months following initial BSSG exposure, compared to controls. Although the average loss of TH^+^ cells was not significant at 4 months, hemispheric differences in TH^+^ cell counts were evident at this time point. When hemispheric comparisons of TH^+^ and Nissl^+^ cell counts were made, hemispheric asymmetry was found to be significantly higher in BSSG-fed animals at 4 months following initial exposure, peaked at 6 months, and significantly dropped at 8 months (TH: F_1,71_ = 166.46, *p*<0.0001, BSSG main effect; F_3,71_ = 33.91, *p*<0.0001, time main effect; F_3,71_ = 39.61, *p*<0.0001, interaction effect) (Nissl: F_1,71_ = 124.51, *p*<0.0001, BSSG main effect; F_3,71_ = 30.12, *p*<0.0001, time main effect; F_3,71_ = 23.99, *p*<0.0001, interaction effect) (Fig 6C and 6E). No significant hemispheric asymmetry was evident at 10 months following initial BSSG exposure.

**Fig 7:**
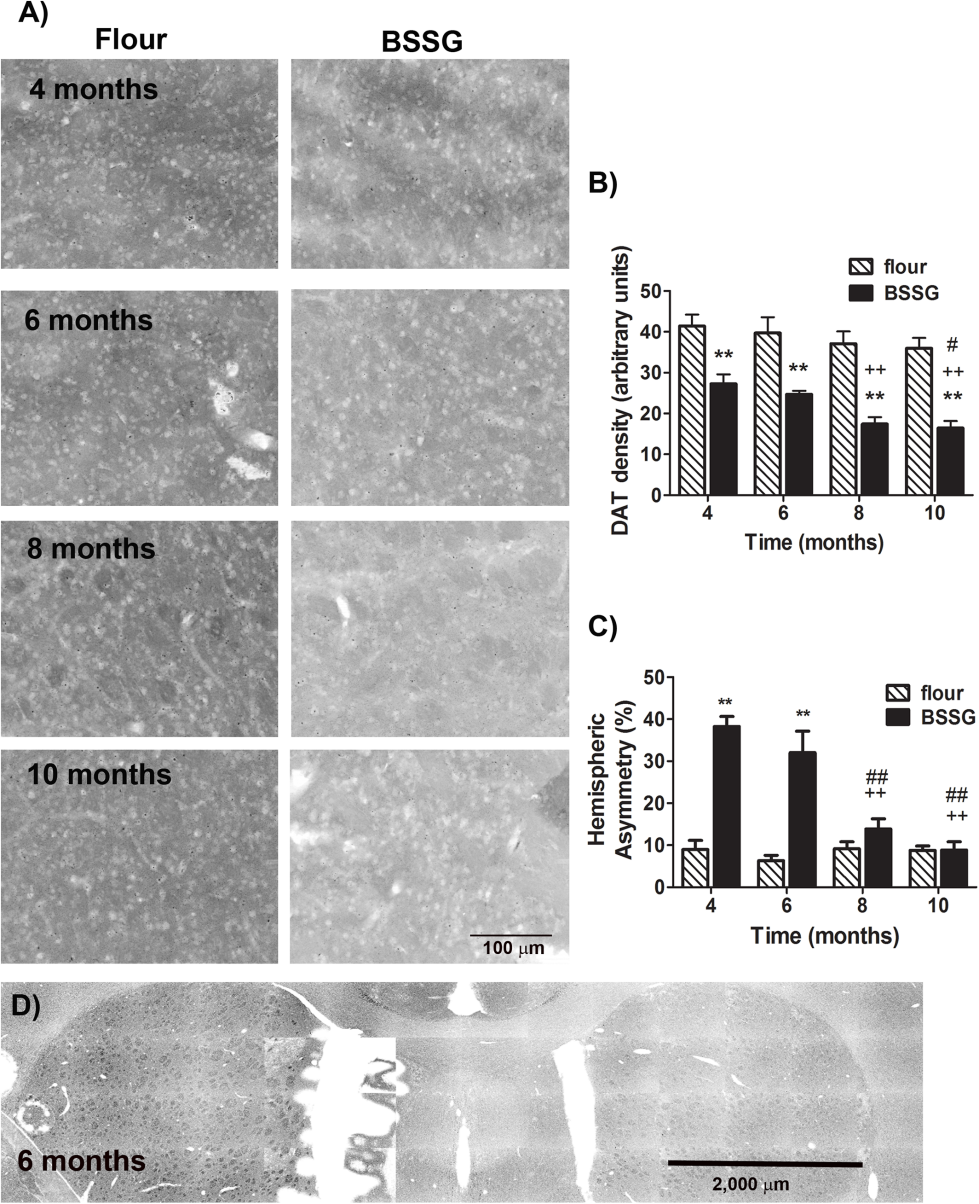
BSSG triggers the progressive loss of striatal DAT. (**A)** Representative fluorescent photomicrographs of dopamine transporter (DAT) immunostaining in the striatum 4, 6, 8, and 10 months following initial BSSG exposure. (**B)** Immunolabeling for DAT was significantly reduced, beginning as early as 4 months following initial BSSG exposure. Each bar represents the mean (± S.E.M., *n* = 9–10) optical density measured in 3 regions across 4 coronal sections through the striatum. (**C)** Asymmetrical loss of DAT immunolabeling in the striatum was observed at 4 and 6 months following initial BSSG exposure, with no significant hemispheric asymmetry observed at 8 or 10 months. Each bar represents the mean (± S.E.M., *n* = 9–10) hemispheric difference, expressed as a percentage of the most populated hemisphere. (**D**) Representative photomicrograph depicting asymmetry in DAT immunolabeling in the striatum 6 months following initial exposure to BSSG. ** sig. diff. from flour control, *p* < 0.001; ++ sig. diff. from 4 months, *p* < 0.001; ## sig. diff. from 6 months, *p* < 0.001; # *p* < 0.05.

Striatal innervation by nigrostriatal neurons was determined by densitometric measurement of DAT immunolabeling in the striatum. DAT immunolabeling was significantly reduced in the striatum following exposure to BSSG, beginning at 4 months following initial BSSG exposure and progressing in a time-dependent manner (F_1,71_ = 185.04, *p*<0.0001, BSSG main effect; F_3,71_ = 9.26, *p*<0.0001, time main effect; F_3,71_ = 1.28, *p* = 0.288, interaction effect) (Fig 7). No significant regional variation was observed within the striatum. Similar to the loss of TH^+^ neurons in the SNc, striatal DAT immunolabeling exhibited hemispheric asymmetry following BSSG exposure, peaking at 4 and 6 months following initial exposure (F_1,71_ = 259.27, *p*<0.0001, BSSG main effect; F_3,71_ = 45.36, *p*<0.0001, time main effect; F_3,71_ = 61.83, *p*<0.0001, interaction effect). By contrast, no significant difference in hemispheric asymmetry was apparent at 8 or 10 months following initial BSSG exposure. Although hemispheric asymmetry was not lateralized to the same hemisphere in all animals, where significant asymmetry was apparent, decreases in nigral and striatal immunolabeling were lateralized to the same hemisphere in each animal.

### Inflammation

**Fig 8:**
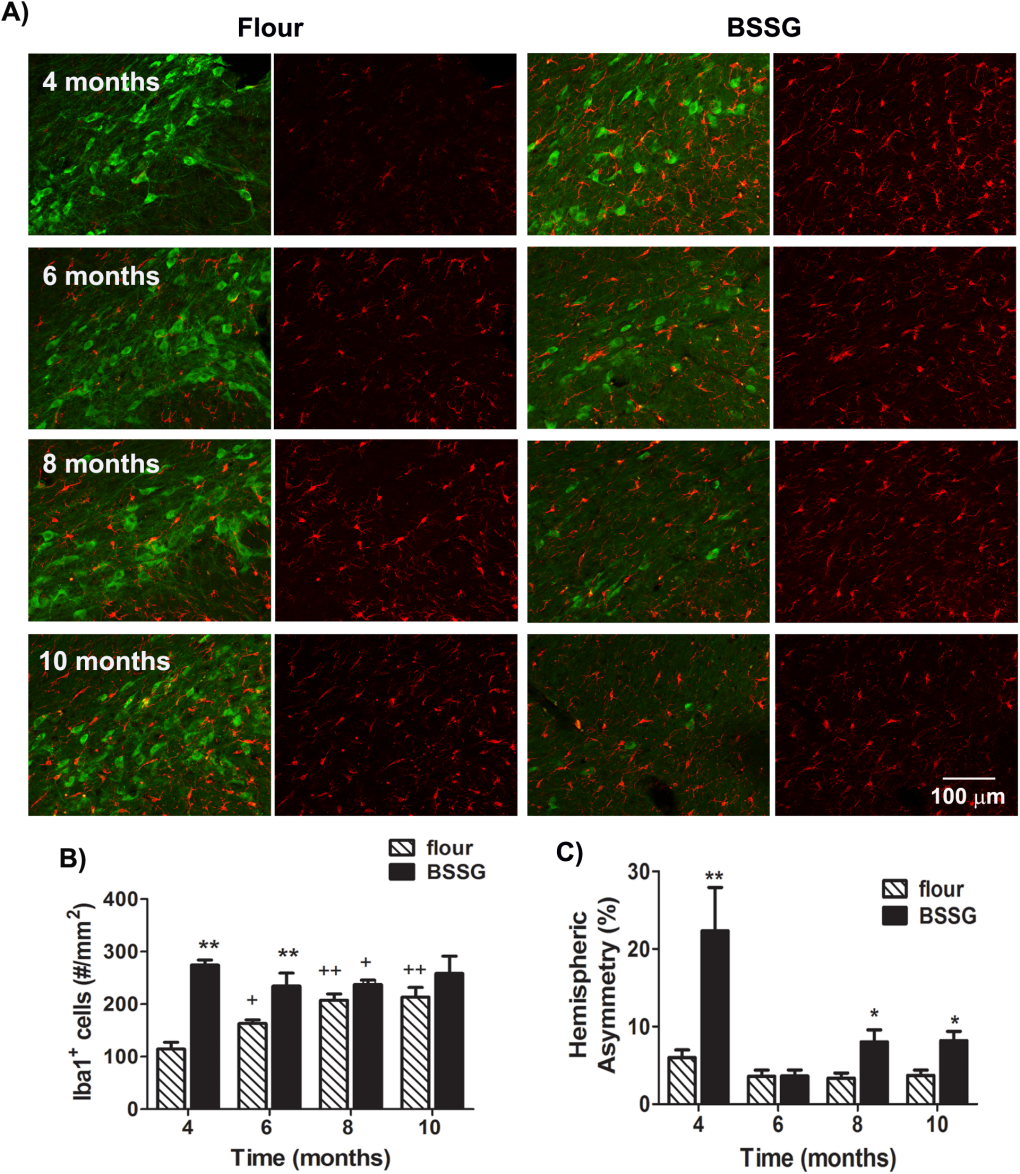
BSSG triggers a progressive inflammatory response. **(A)** Representative fluorescent photomicrographs of Iba1 (red) and TH (green) immunolabeling in the SNc 4, 6, 8, and 10 months following initial BSSG exposure. (**B)** BSSG exposure resulted in a significant elevation in the number of activated microglia in the SN_C_. Each bar represents the mean (± S.E.M., *n* = 9–10) density of Iba1-positive cells counted in the SNc. (**C)** Asymmetric elevations in Iba1 immunolabeling were observed at 4 months and, to a lesser extent, at 8 and 10 months following initiation of BSSG intoxication. Each bar represents the mean (± S.E.M., *n* = 9–10) hemispheric difference in Iba1 density, expressed as a percentage of the most populated hemisphere. ** sig. diff. from flour control, *p* < 0.001; * *p* < 0.05; ++ sig. diff. from 4 months, *p* < 0.001; + *p* < 0.05.

Microglial activation was assessed using immunolabeling for ionized calcium binding adaptor molecule 1 (Iba1), a microglia/macrophage-specific calcium-binding protein known to play a role in regulating the function of microglia, especially in activated microglia [17]. Indications of an inflammatory response appeared early and persisted. BSSG exposure resulted in a significant elevation in the number of Iba1-positive cells in the SN_C_ at all time points (F_1,71_ = 121.09, *p*<0.0001, BSSG main effect; F_3,71_ = 6.03, *p* = 0.001, time main effect; F_3,71_ = 18.54, *p*<0.0001, interaction effect) (Fig 8). Hemispheric asymmetry in Iba–1 immunolabeling was significantly higher in BSSG fed animals at 4 months following initial exposure. While no significant difference in hemispheric asymmetry was observed at 6 months, a slight, but significant, increase in asymmetry was evident at 8 and 10 months following initial exposure to BSSG (F_1,71_ = 99.26, *p*<0.0001, BSSG main effect; F_3,71_ = 51.51, *p*<0.0001, time main effect; F_3,71_ = 26.71, *p*<0.0001, interaction effect).

### α-Synuclein aggregates

**Fig 9:**
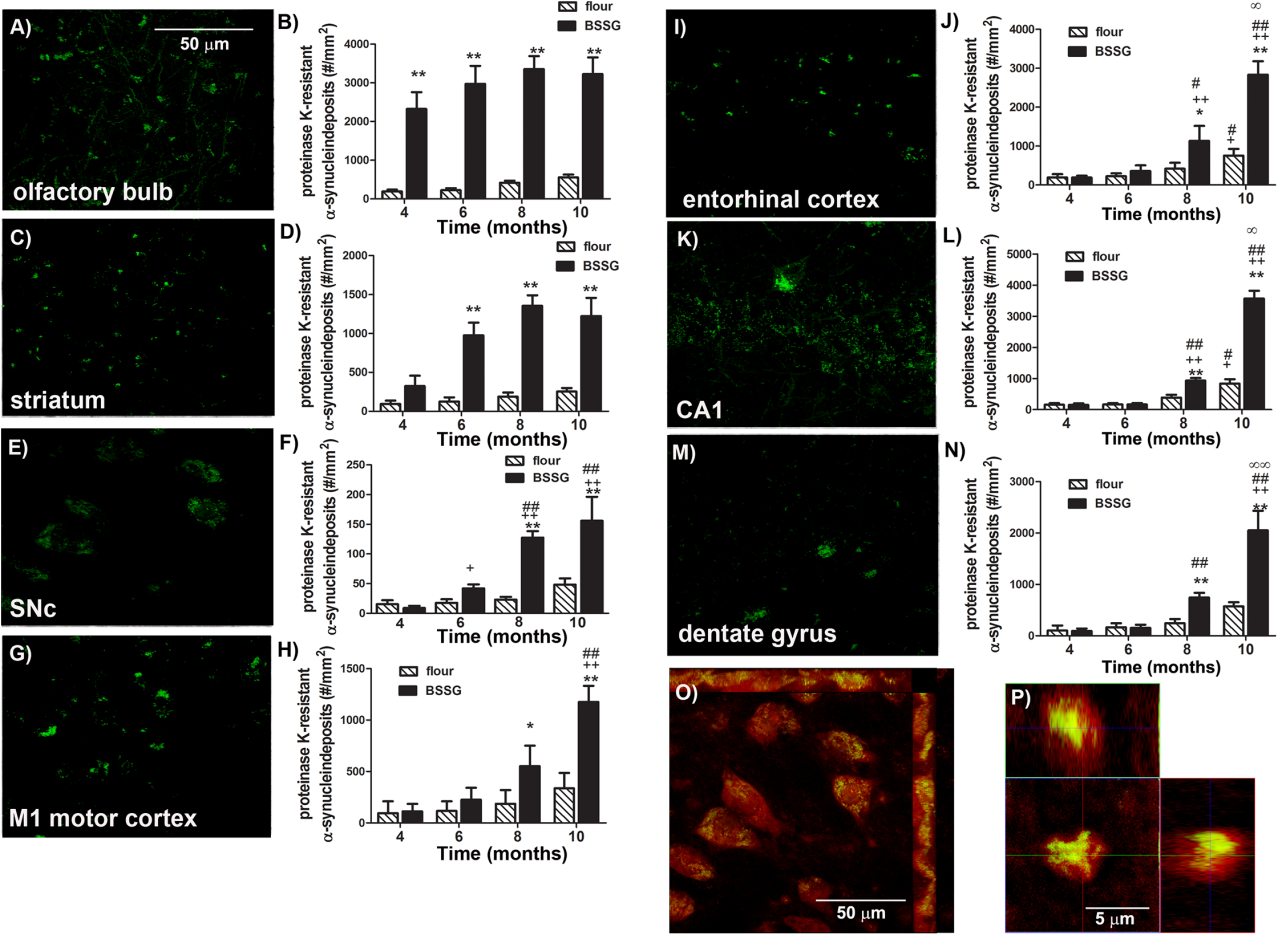
BSSG promotes the accumulation of proteinase K-resistant α-synuclein aggregates. Representative photomicrographs depicting proteinase K-resistant α-synuclein aggregates in the (**A)** olfactory bulb, (**C)** striatum, (**E)** substantia nigra, (**G)** M1 motor cortex, (**I)** entorhinal cortex, (**K)** CA1, and (**M)** dentate gyrus 10 months following initial BSSG exposure. The average density of proteinase K-resistant α-synuclein aggregates appearing in the (**B)** olfactory bulb, (**D)** striatum, (**F)** substantia nigra, (**H)** M1 motor cortex, (**J)** entorhinal cortex, (**L)** CA1, and (**N)** dentate gyrus following BSSG exposure was significantly elevated in a time-dependent manner. Each bar represents the mean (± S.E.M., *n* = 9–10) number of α-synuclein-positive aggregates counted per mm^2.^ (**O)** Representative high magnification (63X) fluorescent photomicrograph of insoluble synuclein aggregates localized within TH-positive neurons. red = TH; green = proteinase K-resistant α-synuclein. (**P**) Representative fluorescent photomicrograph of a proteinase K-resistant α-synuclein deposit stained with Thioflavin S. red = proteinase K-resistant α-synuclein; green = Thioflavin S. ** sig. diff. from flour control, *p* < 0.001; * *p* < 0.05; ++ sig. diff. from 4 months, *p* < 0.001; + *p* < 0.05; # sig. diff. from 6 months, *p* < 0.05.

Lewy bodies are a key pathological feature of PD and contain large amounts of α-synuclein aggregates. Alpha-synuclein is a presynaptic neuronal protein that is linked both genetically and neuropathologically to PD and may contribute to PD pathogenesis in a number of ways. Under pathological conditions, α-synuclein oligomerizes and aggregates into fibrils [18] Sterol gluocosides, derived from cycad, have been shown to directly enhances aggregation and cytotoxicity of α-synuclein *in vitro* [19], while cycad flour triggers the appearance of Lewy body-like synuclein aggregates in rats [10]. The ability to model abnormal α-synuclein accumulation in an animal model is critical to the identification of potential therapeutic targets. Here, we assessed sections for accumulation of proteinase K-resistant α-synuclein immunolabeling in various regions of the brain. Treatment with BSSG was found to promote the accumulation of proteinase K-resistant α-synuclein aggregates in a time-dependent manner. At 4 months following initial exposure, BSSG fed animals exhibited a significant increase in α-synuclein aggregates in the olfactory bulb that persisted throughout the study (F_1,71_ = 899.70, *p*<0.0001, BSSG main effect; F_3,71_ = 18.36, *p*<0.0001, time main effect; F_3,71_ = 7.58, *p* = 0.0002, interaction effect) (Fig 9). Aggregates were not found in any other examined region, at this time point. At 6 months following initial BSSG exposure, synuclein aggregates were significantly higher in the striatum of BSSG-intoxicated animals (F_1,71_ = 516.45, *p*<0.0001, BSSG main effect; F_3,71_ = 57.73, *p*<0.0001, time main effect; F_3,71_ = 42.90, *p*<0.0001, interaction effect). Significant elevations in synuclein deposits were also found, though to a lesser degree, in the SNc at this time point, and increased significantly at 8 and 10 months following initial exposure (F_1,71_ = 138.18, *p*<0.0001, BSSG main effect; F_3,71_ = 50.69, *p*<0.0001, time main effect; F_3,71_ = 30.90, *p*<0.0001, interaction effect). These deposits co-labeled with ubiquitin and were localized within dopaminergic neurons, although larger extracellular aggregates were also present, particularly in the latest time point, when the majority of dopaminergic neurons were lost. A subset of aggregates stained positive for Thioflavin S. No significant increase in cortical or hippocampal aggregates was noted until 8 and 10 months, at which time, BSSG induced a significant elevation in the number of proteinase K-resistant synuclein deposits in primary motor cortex (M1), entorhinal cortex, CA1 and dentate gyrus (M1: F_1,71_ = 169.33, *p*<0.0001, BSSG main effect; F_3,71_ = 152.00, *p*<0.0001, time main effect; F_3,71_ = 78.73, *p*<0.0001, interaction effect) (EC: F_1,71_ = 151.42, *p*<0.0001, BSSG main effect; F_3,71_ = 149.07, *p*<0.0001, time main effect; F_3,71_ = 47.43, *p*<0.0001, interaction effect) (CA1: F_1,71_ = 249.77, *p*<0.0001, BSSG main effect; F_3,71_ = 351.81, *p*<0.0001, time main effect; F_3,71_ = 162.04, *p*<0.0001, interaction effect) (DG: F_1,71_ = 178.41, *p*<0.0001, BSSG main effect; F_3,71_ = 237.23, *p*<0.0001, time main effect; F_3,71_ = 94.08, *p*<0.0001, interaction effect).

### Grp78 (BiP)

**Fig 10:**
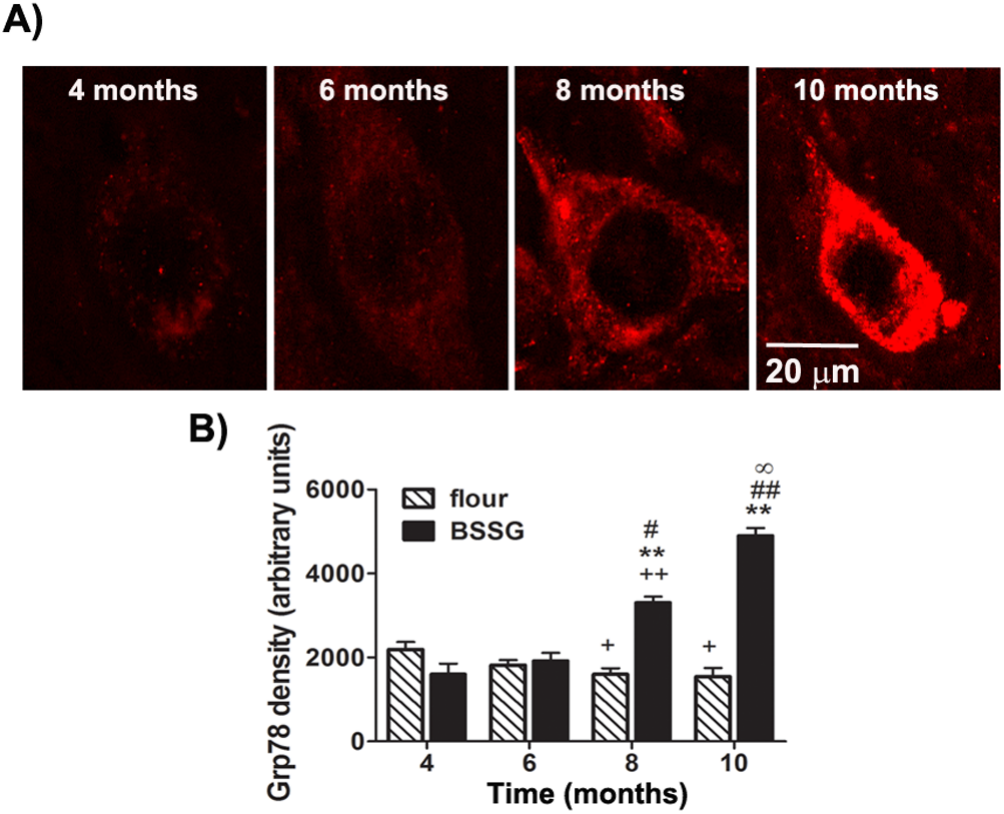
BSSG triggers the progressive increase in nigral Grp78. (**A)** Representative fluorescent photomicrographs of Grp78 immunostaining in the SNc 4, 6, 8, and 10 months following initial BSSG exposure. (**B)** Intracellular immunodensity of Grp78 was significantly reduced in controls at 8 and 10 months, as compared to 4 months. In BSSG-fed animals, Grp78 immunolabeling was significantly elevated at 8 and 10 months following initial toxin exposure, as compared to controls. Each bar represents the mean (± S.E.M., *n* = 9–10) optical density measured in 10 cells/section across 4 coronal sections through the SNc. ** sig. diff. from flour control, *p* < 0.001; ++ sig. diff. from 4 months, *p* < 0.001; + *p* < 0.05; ## sig. diff. from 6 months, *p* < 0.001; # *p* < 0.05; ∞ sig. diff. from 8 months, *p* < 0.05.

Glucose-regulated protein 78 (Grp78) is a molecular chaperone, required for endoplasmic reticulum integrity and stress-induced autophagy. It plays a central role in the regulation of the unfolded protein response, an attempt by the cell to protect itself against the toxic effects of misfolded proteins [20–22]. Grp78 up-regulation has become a classical marker for ER stress and UPR induction. Here, we examined immunolabeling for Grp78 in the SN and striatum, as a marker of ER stress. In control animals, optical density of intracellular Grp78 immunolabeling in the SNc was slightly, but significantly, reduced over time, with a 27% and 30% reduction at the 8 and 10 month time points, respectively, as compared to 4 months into the study (F_1,71_ = 465.25, *p*<0.0001, BSSG main effect; F_3,71_ = 118.23, *p*<0.0001, time main effect; F_3,71_ = 278.23, *p*<0.0001, interaction effect) (Fig 10). This is consistent with reports of an age-related decline in chaperone concentrations [23]. By contrast, BSSG-fed animals were found to display and increase in immunolabeling for Grp78, with significantly higher immunodensity at 8 and 10 months following initial toxin exposure, relative to controls.

### Synaptophysin

**Fig 11:**
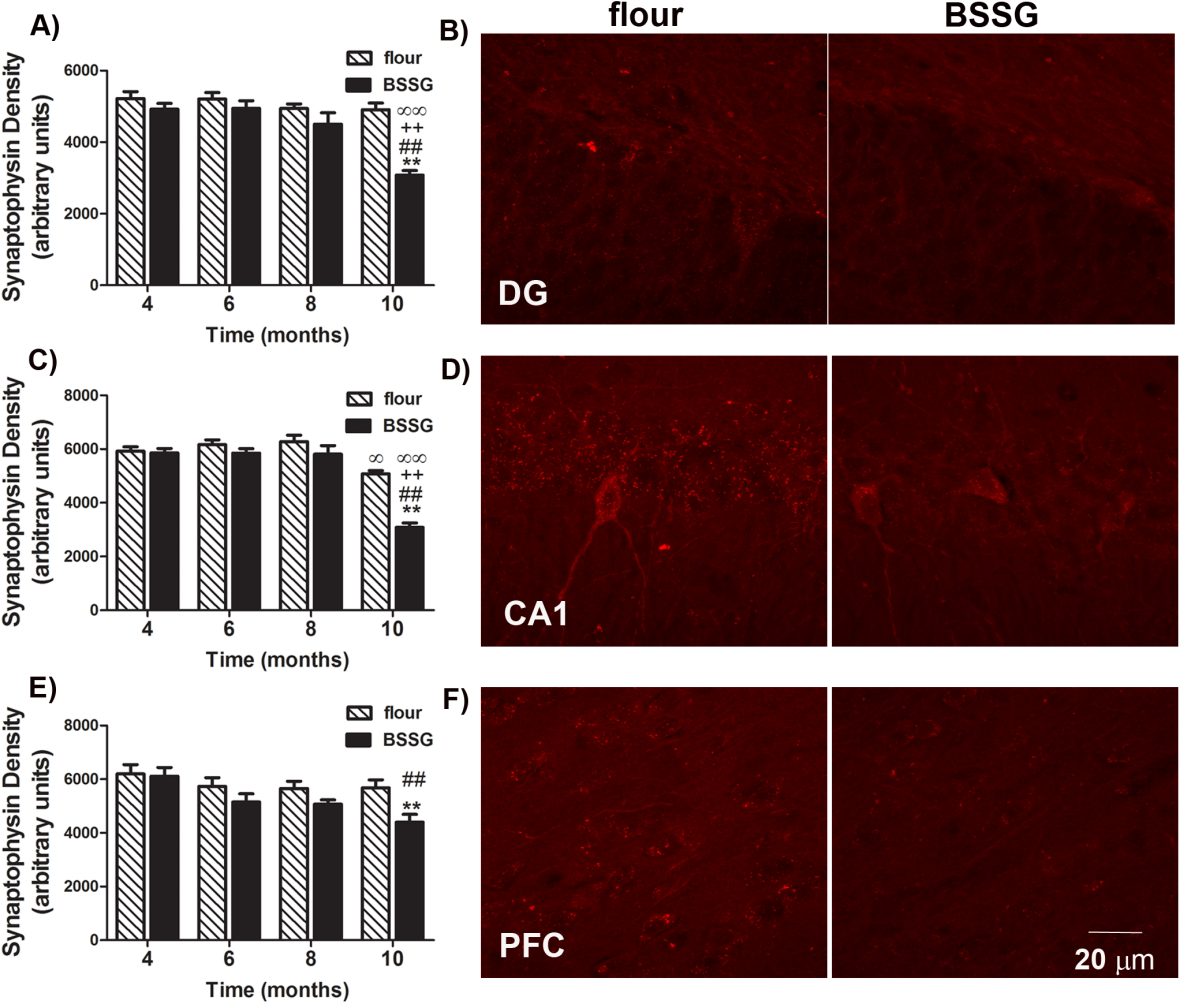
BSSG triggers hippocampal and cortical synaptic atrophy. Representative fluorescent photomicrographs depicting immunolabeling for the synaptic protein, synaptophysin, in the (**B**) DG, (**D**) CA1 region of the hippocampus, and (**F**) prefrontal cortex of flour- and BSSG-treated animals 10 months following initial BSSG exposure. Immunolabeling for synaptophysinwas significantly reduced in the (**A)** DG and **(C)** CA1 region of the hippocampus, as well as **(E)** the prefrontal cortex at 10 months following initial BSSG exposure, as compared to controls. In the CA1 region, there was a slight, but significant, decline in synaptophysin immunodensity observed in controls, relative to earlier time points. Each bar represents the mean (± S.E.M., *n* = 9–10) optical density measured. ** sig. diff. from flour control, *p* < 0.001; ++ sig. diff. from 4 months, *p* < 0.001; ## sig. diff. from 6 months, *p* < 0.001; ∞∞ sig. diff. from 8 months, *p* < 0.001; ∞ *p*< 0.05.

In order to estimate degenerative axonal pathology in the hippocampus and cortex, we examined the synaptic protein, synaptophysin. No significant changes in synaptophysin immunodensity were observed until 10 months following initial BSSG exposure. At this time, BSSG-fed animals were found to have a significant reduction in immunolabeling of the hippocampal DG and CA1, as well as prefrontal cortex (DG: F_1,71_ = 172.61, *p*<0.0001, BSSG main effect; F_3,71_ = 83.40, *p*<0.0001, time main effect; F_3,71_ = 52.04, *p*<0.0001, interaction effect) (CA1: F_1,71_ = 140.04, *p*<0.0001, BSSG main effect; F_3,71_ = 259.56, *p*<0.0001, time main effect; F_3,71_ = 51.19, *p*<0.0001, interaction effect) (PFC: F_1,71_ = 64.58, *p*<0.0001, BSSG main effect; F_3,71_ = 33.05, *p*<0.0001, time main effect; F_3,71_ = 9.92, *p*<0.0001, interaction effect) (Fig 11).

## Discussion

In this study, we provide an in-depth assessment of a novel, progressive animal model of PD that exhibits many characteristic features of the disease. Among these is the pre-motor appearance of olfactory deficits. Indeed, hyposmia is one of various non-motor symptoms characteristic of PD [24], occurring as early as 5–7 years before the onset of motor symptoms. Upon autopsy, PD brains display atrophy and synucleinopathy in the olfactory bulb [25] and diffusion tensor imaging reveals structural abnormalities that strongly correlate with olfactory function [26]. The early identification of these olfactory abnormalities may serve as a predictive biomarker for identifying early, pre-motor PD [26, 27]. This early diagnosis will be critical for neuroprotective therapies, which will require early intervention for maximal effectiveness [28]. In this way, the development of disease-modifying treatments, and identification of early biomarkers, go hand in hand. With this in mind, effective pre-clinical screening of neuroprotective candidates will require, not only a progressive model, but also one that expresses a specific and selective biomarker, such as olfactory dysfunction, to serve as a corollary time point for intervention. Unfortunately, most available rodent models of PD either do not display olfactory dysfunction, or the origin of the deficits may not reflect that found in PD [29]. Interestingly, transgenic mouse models overexpressing α-synuclein do exhibit olfactory deficits, which are associated with the appearance of synuclein inclusions in the olfactory bulb [30, 31]. This is consistent with the Braak staging hypothesis [32], which describes the appearance of inclusion body formations in the olfactory bulb during early pre-symptomatic stages. Here, we have demonstrated the appearance of olfactory deficits in BSSG-fed rats at a time when no significant locomotor deficits were apparent. Furthermore, the first appearance of abnormal α-synuclein aggregates, in these animals, was in the olfactory bulb, also prior to the development of motor deficits. Thus, the BSSG model may provide a useful tool for screening disease-modifying therapies in the early, pre-motor stages.

The development of effective neuroprotective strategies has historically been hampered by limitations in the majority of current animal models of PD, which fail to mimic the progressive nature of the disease. Rather than targeting acute responses to the initiating factor, effective therapeutic interventions will need to target the self-perpetuating cascade of pathological events that continues long after the initial insult occurs. Effective screening of such therapies will require an animal model capable of recapitulating disease progression in the absence of the initiating event/toxin. Here, chronic exposure to BSSG resulted in the progressive loss of nigrostriatal dopaminergic neurons that did not begin until after toxin exposure was terminated. Thus, at the time BSSG feeding was completed, no significant loss of TH^+^ neurons in the SNc was apparent. Rather, neuronal loss occurred gradually over the ensuing months, approaching a 70% loss of nigral neurons by 10 months following the initial exposure to BSSG. The ability of the BSSG model to reflect the progressive nature of the disease and its complexity make it an ideal tool for screening neuroprotective therapies for PD. Indeed, its protracted development permits intervention with therapeutic candidates at more clinically-relevant time points, such as the first appearance of olfactory or motor deficits. Further, it also allows therapies to be administered in the absence of the initiating factor, thereby eliminating confounds associated with possible direct interaction with the toxin, itself, rather than the resulting disease process.

Another unique characteristic feature of this model is its asymmetric onset. In PD, the lateralized onset of the disease is a common occurrence [33–36], and is listed as part of the classical staging system originally proposed by Hoehn and Yahr [37], in which stage I disease is defined by unilaterality of motor features [38]. Here, we report locomotor asymmetry in the early stages of BSSG intoxication, accompanied by the asymmetric loss of dopaminergic neurons in the SNc and DAT immunodensity in the striatum. Inflammation, the earliest pathological sign observed, also displayed an asymmetric appearance at 4 months following initial toxin exposure. While this asymmetry declined significantly, it reappeared, albeit to a lesser extent, at 8 and 10 months. This may reflect a slight delay in the inflammatory response of one hemisphere relative to the other. A longtitudinal imaging assessment may be required to confirm this. While the underlying mechanism for such laterality remains a mystery, understanding these hemispheric differences in susceptibility may provide critical clues to further our understanding of PD pathogenesis.

Although the loss of dopamine in the nigrostriatal system is the main pathological hallmark of PD, the disease progresses to involve other regions of the brain, including the hippocampus and cortex, and other neurotransmitter systems, including deficits in cholinergic transmission [32, 39]. Consequently, the majority of PD patients go on to develop dementia within 12 years of diagnosis [40]. Parkinson's disease dementia (PDD) is often associated with more rapid motor and functional decline and increased mortality and is a frequent cause of institutionalization in these patients [41]. Effective treatment of PDD remains an important unmet medical need, one that cannot be addressed without appropriate animal models. Here, BSSG intoxication resulted in the development of cognitive deficits at 10 months following initial exposure. Disrupted spontaneous alternation in the T-maze test and impaired performance in the RAM suggest the late-stage development of working memory deficits, similar to that observed in PDD [42] This disruption in reference and working memory performance was accompanied by the appearance of proteinase K-resistant synuclein aggregates in the hippocampus and cortex, along with reductions in synaptophysin density, suggesting a regional progression of pathology, similar to that reported in PD patients [32, 43]. Indeed, the reduction in synaptophysin density observed here may relate to axonal pathology and loss of hippocampal innervation observed in patients with PDD [44, 45]. A more in-depth assessment of hippocampal and cortical pathological hallmarks, as well as effects in other neurotransmitter systems, is currently underway. Based on the findings reported here, the BSSG model of PD may provide an effective model for screening treatments for PDD.

The main pathologic hallmark of PD involves the accumulation of intraneuronal aggregates of the protein, α-synuclein forming Lewy bodies, a toxic event in the development of PD. Sterol gluocosides, derived from cycad, have been shown to directly enhance aggregation and cytotoxicity of α-synuclein in vitro[19], while cycad flour triggers the appearance of Lewy body-like synuclein aggregates in rats[10].Autopsy-based studies by Braak and coworkers demonstrate a region to region disease progression in six phases (Braak stages), starting in the olfactory bulb and gut and progressing along interconnected regions towards the caudate and substantia nigra. In some cases the pathology spreads further to mesocortical and neocortical areas [32, 46]. The extent of this synuclein pathology correlates with the nature and severity of clinical symptoms [47, 48]. A similar pattern was observed in the brains of BSSG-intoxicated rats. Here, we show that BSSG first triggers the appearance of proteinase K-resistant synuclein aggregates in the olfactory bulb, coincident with the initiation of olfactory deficits. This is strikingly similar to Braak stage 1 and 2, which are pre-motor stages. These synuclein aggregates then began to appear in the midbrain, around the time significant locomotor deficits developed in these animals. This midbrain pathology and motor phenotype are similar to Braak stage 3 and 4. Finally, at the later time points studied, BSSG resulted in the spread of synuclein pathology to hippocampal and cortical regions, coincident with a decline in cognitive function. This is similar to Braak stage 5 and 6. The progressive appearance and regional spread of synucleinopathy following BSSG, may make this a good candidate for screening disease-modifying therapies targeting these processes.

The BSSG model of PD has its origins in human disease. Epidemiological evidence indicates a high incidence of ALS/PDC in the Chamorro people of Guam, first characterized in the 1950s [1]. This syndrome includes features of ALS, PD, and dementia, combined to varying degrees. Indeed, there is extensive clinical, pathologic and genetic heterogeneity in this population. Analysis of Guam post-mortem tissue has identified the appearance of neurofibrillary tangles, alpha synuclein deposits, and beta amyloid plaques, along with abnormalities in Lrrk2, ubiquitin, and TDP–43 [2,3]. The relative involvement of each appears to be dependent on the predominant clinical features presented. Although still controversial, there is a strong positive correlation between Guamanian ALS-PDC and the chronic consumption of phytotoxins found in washed cycad seed [4,5], used as a primary food source during times of famine. The unusually high, but isolated incidence, combined with a striking recent decline, supports a strong role for environmental factors. However, other factors may also be involved, including a variety of genetic mutations recently described in this population [6], which may increase genetic risk. Such a combination of genetic and environmental factors may contribute to phenotypic variability. Irrespective of the causal role cycad flour consumption may have played in this syndrome, its investigation has led to the identification of various water-insoluble phytosterol glucoside components, of which the largest fraction, BSSG, has been demonstrated to be neurotoxic both in vitro and in vivo [7]. Interestingly, the response to this toxin is somewhat species-dependent. In earlier work with cycad flour, distinct phenotypes were found to develop in mice, as compared to rats. When mice are chronically exposed to cycad, they develop a predominantly Amyotrophic Lateral Sclerosis (ALS) phenotype, with profound motor neuron pathology and only mild parkinsonian indices appearing in the later stages [8]. By contrast, no motor neuron loss is evident in rats [10]. Such species variability has been reported for other parkinsonism-inducing neurotoxins, such as 1-methyl-4-phenyl–1,2,3,6-tetrahydropyridine (MPTP), which causes parkinsonism in humans and other primates, but not in rats. Susceptibility in mice is somewhat intermediate but varies among strains [49, 50]. Further understanding of the mechanisms underlying these species differences may contribute to our understanding of PD pathogenesis.

A further understanding of the mechanism of action will be critical for the full optimization and utilization of this model and may also shed some light on the pathogenic processes involved in ALS-PDC. Although the mechansims underlying the pathology remains unknown, alterations in lipid homeostasis may be a factor. Maintenance of lipid homeostasis is increasingly recognized as a crucial factor for normal neuronal function. Modulation of cerebral lipid metabolism or transport may be linked to neurodegenerative pathways in PD [51]. Of particular interest is the finding that α-synuclein is a lipid binding protein and that it deposits with lipids associated with Lewy bodies and neuromelanin in PD tissues [52–54]. Dietary plant sterols are structurally very similar to cholesterol and readily cross the blood-brain barrier to accumulate in the brain [55] where they act as Liver X receptor ligands to regulate cholesterol homeostasis [56, 57]. Liver X receptor β (LXRβ) is express in microglia and astroglia of the SN and have been found to play an important role in dopaminergic neuron survival [56, 58]. Disruptions in LXRβ signalling by BSSG could lead to alterations in cholesterol homeostasis, activation of microglia, accumulation of synuclein aggregates and, ultimately, nigral cell loss. Efforts are currently underway to elucidate this potential mechanism of action.

In summary, this BSSG rat model of PD is characterized by the progressive loss of nigral dopaminergic neurons, displays prodromal olfactory deficits, an asymmetric onset, levodopa-responsive locomotor deficits, a progressive spread of synuclein pathology, and late-stage cognitive deficits. Efforts to develop a neuroprotective therapy for PD have been severely hindered by the lack of good animal models capable of accurately predicting effective neuroprotective agents in humans. Toxic models offer some of the hallmarks of PD, while genetic models offer others, but no one model recapitulates all the features of PD. The BSSG model, through its progressive nature and replication of multiple key parkinsonian features, may provide a valuable tool for screening neuroprotective candidates. Delineating the mechanism of action may also provide critical clues to help deepen our understanding of PD pathogenesis.

## Supporting Information

S1 ARRIVE Checklist ARRIVE Checklist. (PDF)

S1 Fig BSSG does not trigger weight loss. Animals were weighed each week as part of regular health assessments. BSSG feeding did not trigger weight loss in any of the animals tested and there were no significant differences in weight gain over the course of the study.
(TIF)

## Abbreviations

BSSG: β-sitosterol β-D-glucoside
