# Supplementary figures and images for: The Progressive BSSG Rat Model of Parkinson's: Recapitulating Multiple Key Features of the Human Disease
Source: PLoS One. 2015 Oct 6;10(10):e0139694. doi: 10.1371/journal.pone.0139694 (PMC4595214; doi:10.1371/journal.pone.0139694)

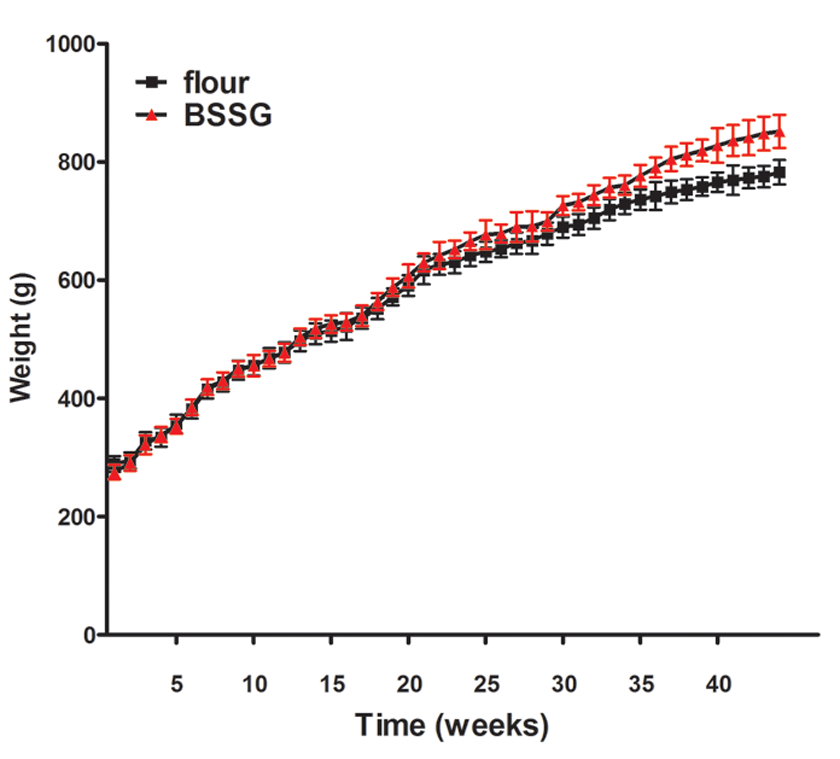

Supplement: S1 Fig — Animals were weighed each week as part of regular health assessments. BSSG feeding did not trigger weight loss in any of the animals tested and there were no significant differences in weight gain over the course of the study. (TIF) [file pone.0139694.s002.tif]
